# Ethylene Signaling in Regulating Plant Growth, Development, and Stress Responses

**DOI:** 10.3390/plants14030309

**Published:** 2025-01-21

**Authors:** Xiaoyi Wang, Hongyi Wen, Andrey Suprun, Hongliang Zhu

**Affiliations:** 1 The College of Food Science and Nutritional Engineering, China Agricultural University, Beijing 100083, China; s20233061281@cau.edu.cn (X.W.); 2021306120117@cau.edu.cn (H.W.); 2 Federal Scientific Center of the East Asia Terrestrial Biodiversity, Far Eastern Branch of the Russian Academy of Sciences, 690022 Vladivostok, Russia; suprun@biosoil.ru

**Keywords:** ethylene signaling, plant growth, stress response, epigenetic modifications, respiration

## Abstract

Ethylene is a gaseous plant hormone that plays a crucial role in coordinating various physiological processes in plants. It acts as a key mediator, integrating both endogenous developmental cues and external environmental signals to regulate a wide range of functions, including growth, fruit ripening, leaf abscission, and responses to stress. The signaling pathway is initiated when ethylene binds to its receptor. After decades of research, the key components of ethylene signaling have been identified and characterized. Although the molecular mechanisms of the sensing of ethylene signal and its transduction have been studied extensively, a new area of research is how respiration and epigenetic modifications influence ethylene signaling and ethylene response. Here, we summarize the research progress in recent years and review the function and importance of ethylene signaling in plant growth and stress responses. In addition, we also describe the current understanding of how epigenetic modifications regulate ethylene signaling and the ethylene response. Together, our review sheds light on the new signaling mechanisms of ethylene.

## 1. Introduction

Ethylene, an important plant hormone, plays a vital role in plant growth and stress response [1]. Ethylene is not only involved in the regulation of plant growth and development, such as root growth, leaf and flower abscission, and fruit ripening [1,2], but it also plays a vital role in the response of plants to various biotic and abiotic stresses [3,4,5]. Since the first report of ethylene biosynthesis and its regulatory mechanism was published in 1984 [6], many studies have gradually deepened our understanding of its function [7,8]. Previous studies have mainly focused on the ethylene synthesis pathway [9,10,11], but with the development of molecular biology technology, we have a more detailed understanding of the ethylene signaling pathway [12,13,14]. Ethylene is produced by the activity of 1-amino-1-carboxycyclopropane (ACC) synthase and ACC oxidase [15] and ultimately affects the expression of downstream genes through the EIN2/EIN3/EIL1 signaling pathway, thereby regulating the physiological response of plants [16,17]. In addition, there are complex interactions between ethylene and other plant hormones such as auxins, which are of great significance for plants to adapt to environmental changes [17,18]. Therefore, to better understand the ethylene signal transduction pathway in plants, we summarized its metabolism and signaling receptors, as well as its functions in regulating plant growth, development, and biotic and abiotic stress responses. It is worth emphasizing that ethylene signaling mainly pertains to the process in which ethylene molecules are perceived by plant cells and then undergo signal transmission and transduction through a series of specific molecular mechanisms. Typically, it does not cover the biosynthesis steps of ethylene, yet it is closely associated with and mutually influences biosynthesis.

Ethylene is closely related to respiration in plant growth, development, and environmental adaptation [19,20,21,22]. Respiration is the core of plant cell energy metabolism, occurs in mitochondria, and involves electron transport chain reactions. When the fruit matures, ethylene production increases greatly and the respiration rate increases simultaneously. The two are closely related and intertwined. Ethylene not only affects respiration rate, but also affects fruit ripening speed and its quality by regulating its own production and signaling [22,23]. For example, while ACS (1-aminocyclopropane-1-caroboxylic acid synthase) protein promotes ethylene synthesis, its high expression may provide a substrate for mitochondrial aerobic respiration, reflecting the interaction between ethylene and mitochondria in fruit ripening [24]. The AOX (Alternative Oxidase) pathway is related to ethylene in the climacteric respiration of tomato fruits, while there are less data on the COX (Cytochrome Oxidase) pathway [25,26]. When plants respond to hypoxic stress, ethylene, which is also related to respiration, can trigger signal transduction, regulate electron transport, ROS (reactive oxygen species) production, and affect metabolic remodeling [27,28,29]. However, the precise regulation mechanism of ethylene signaling on respiration and mitochondrial function still needs to be further studied.

In addition, epigenetic modifications also play an important role in ethylene signaling [30]. Epigenetic mechanisms such as DNA methylation, histone modification, and non-coding RNAs influence the expression of genes associated with ethylene signaling, thereby finely regulating its activity. Studies have shown that epigenetic modifications can respond to environmental changes, dynamically regulate ethylene signaling, and further enhance plant adaptability to the environment [31]. For example, under low-temperature stress, the DNA methylation status of ethylene signal transduction-related genes in tomato fruits changes, affecting gene expression levels and thereby regulating the fruit ripening process [32]. However, the specific regulatory mechanisms of epigenetic modifications in ethylene signaling and their interactions with other signaling pathways are still unclear and require further exploration.

This review aims to systematically describe the role of ethylene in plant growth and development, stress response, and its regulatory mechanism, summarize current research results, and point out the directions of future research. Through an in-depth analysis of the regulatory network in the ethylene signaling pathway, we hope to reveal how ethylene accurately regulates the response of plants to environmental changes and provide a theoretical basis and technical support for improving crop adaptability and stress resistance. In the review, the functions of ethylene in a variety of processes are expounded, which are primarily founded on the responses detected in mutants or transgenic plants.

## 2. Ethylene Biosynthesis and Signaling in Plants

**Figure 1:**
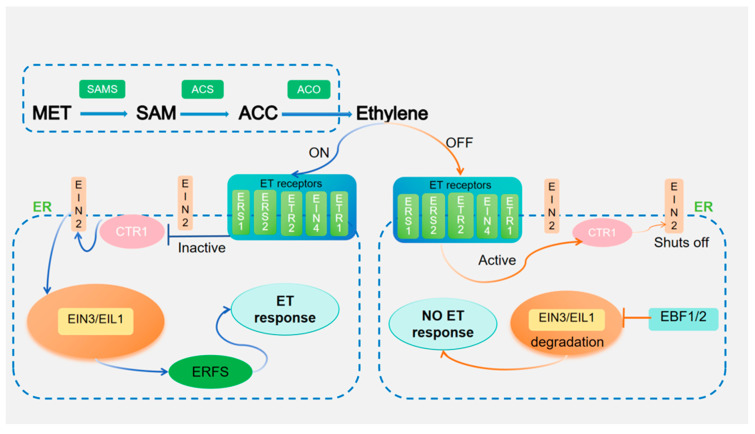
Ethylene synthesis and signal transduction pathways in plants. Without ethylene, its receptor activates CTR1. CTR1 phosphorylates and inhibits EIN2, a membrane protein on the endoplasmic reticulum. EIN2 targets EIN3 and EIL1 to the protease body, blocking transcription. In ethylene’s presence, receptors bind ethylene-inactivating CTR1. It enhances EIN3/EIL1 activity. Ethylene stabilizes EIN3/EIL1 and controls the transcription of ERFs. MET: Methionine; SAM: S-adenosyl-L-methionine; ACC: 1-amino-1-carboxycyclopropane; ACS: ACC synthase; ACO: ACC oxidase; EIN: Ethylene insensitive; ETR1/2: EIN2-Targeting protein 1/2; ERS1/2: Ethylene response sensor 1/2; CTR1: Constitutive triple response 1; EFRS: Ethylene-responsive factors; EBF1/2: EIN3-Binding F-BOX Protein 1 and 2.

After extensive research, the ethylene biosynthesis pathway has become more apparent (Figure 1). The precursors for ethylene synthesis in plants include S-adenosyl-l-methionine (SAM) and 1-amino-1-carboxycyclopropane (ACC) [6]. The ethylene biosynthesis pathway consists of three enzyme-catalyzed reactions: methionine (Met) is catalyzed by methionine adenylyl transferase to form SAM; SAM is catalyzed by ACC synthase (ACS) to form ACC, which simultaneously forms 5′-methylthioadenosine (MTA), and then passes through the Yang cycle to synthesize new methionine (Met). Finally, ACC is catalyzed by ACC oxidase (ACO) to synthesize ethylene [16,33,34]. ACS and ACO are two important rate-limiting enzymes involved in ethylene synthesis.

Ethylene is detected by a group of receptors located on the membrane of the endoplasmic reticulum (ER). In Arabidopsis, the plant hormone ethylene is detected by a group of receptors known as ETHYLENE RESPONSE1/2 (ETR1/2), ETHYLENE RESPONSE SENSOR1/2 (ERS1/2), and ETHYLENE INSENSITIVE4 (EIN4) [35]. These receptors play a suppressive role in ethylene signaling; they are also associated with two-component histidine kinase receptors [36]. In the absence of ethylene, the receptor family recruits Raf-like kinase constitutive triple response 1 (CTR1) to phosphorylate the C-terminal domain of EIN2 [14]. Then, the F-box proteins EIN2-Targeting Protein 1 and 2 (ETP1 and ETP2) degrade the phosphorylated EIN2 to block the downstream transmission of ethylene signaling with EIN3-Binding F-BOX Protein 1 and 2 (EBF1 and EBF2). EBF1/2 interacts with EIN3/EIL1 and regulates their stability through a 26S proteasome degradation pathway [37]. EIN3/EIL1 is degraded by ubiquitinated proteasome, hence the ethylene signaling pathway shuts down [38].

On the other hand, when ethylene is present, it binds to the receptor and inactivates both the receptor and CTR1, and the phosphorylation of EIN2 is inhibited. Without phosphorylation, EIN2 cannot be degraded by ETP1/2 but undergoes its own cleavage. Then, the cleaved EIN2-C-terminus moves to the nucleus, which activates the downstream EIN3/EIL1 transcription cascade and opens up the ethylene signaling pathway [39]. EIN3 can directly bind to the promoter regions of Ethylene Response Factors (ERFs), making for the modulation of downstream gene expression in response to a range of environmental stresses [40]. ERFs are transcription factors unique to plants. ERFs serve as regulatory elements within the ethylene signaling pathway, exerting impact over ethylene and stress-related responses [41,42].

Ethylene biosynthesis is the upstream part of ethylene action, and ethylene signaling is the downstream part, whose research started late and progressed fast, and the biological effects of ethylene are believed to be realized through the ethylene signaling pathway. Currently, a relatively complete signaling pathway has been formed in the model crop Arabidopsis [14], ethylene → ethylene receptor ETR family → CTR family → EIN2 → EIN3/EIL → ERFs → ethylene response-related gene expression.

## 3. Ethylene Signaling Regulates Plant Growth and Development

**Figure 2:**
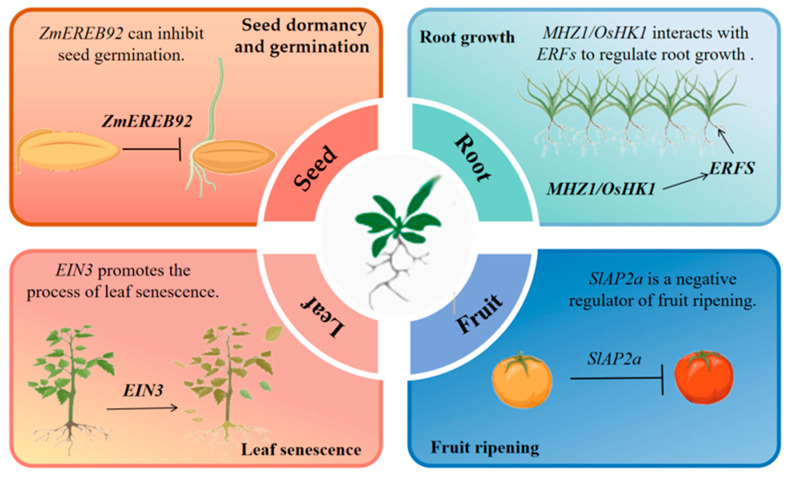
Ethylene signaling regulates plant development. These are some typical examples to illustrate the important role of ethylene signaling in plant growth and development.

In addition to the above effects, ethylene signal transduction regulatory receptors play multiple roles in plant growth and development. Fruit ripening, root development, and seed germination are just a few of the activities that are greatly impacted by ethylene signal transduction in plants. These are some typical examples to illustrate the important role of ethylene signaling in plant growth and development (Figure 2). However, the actual situation is far more complex than this. Furthermore, we must emphasize that other aspects of ethylene’s involvement, such as the measurement of ethylene production and the application of inhibitors, are outside the scope of this review.

### 3.1. Seed Germination and Dormancy

Dormancy allows plant seeds to arrange time for germination when environmental conditions become conducive to the survival and growth of seedlings [43,44]. Ethylene plays a crucial role in breaking the dormancy of many species and can promote the germination of dormant seeds [45]. ERF genes are significantly involved in the ethylene response pathway and the control of seed germination [46,47]. Premature seed germination and accelerated hook development in dark-grown seedlings are the effects of overexpressing the *SlERF2* gene in transgenic tomato lines, exhibiting heightened ethylene sensitivity [48]. *ZmEREB92* inhibits ethylene signaling and maize starch mobilization, which negatively affects seed germination in corn [49]. During the process of seed imbibition and germination, the ethylene receptors, specifically *CpETR2A* and *CpETR2B*, were observed to be activated. This observation provides compelling evidence for the pivotal and favorable function that ethylene signaling plays in promoting the germination of squash seeds [50].

### 3.2. Root Growth

The root serves as the primary structure through which plants uptake water and nutrients, with its growth being intricately controlled by ethylene. The sensing of soil compaction signals by plant roots is mediated by ethylene [51]. ET influences root growth and development through its signaling pathway, including elongation of primary roots, formation of lateral roots, creation of a suitable microenvironment for root stem cells, and stimulation of root hair growth [52]. However, the molecular mechanism is not fully understood.

In the process of root elongation, the effect of ethylene exhibits dose-dependent characteristics [53]. Low concentrations stimulate elongation, while high concentrations inhibit it. This effect results from ethylene’s regulation of cell wall relaxation and gene expression related to cell elongation, such as inducing Expansin Genes like *AtEXP7* and *AtEXP18* [54]. In lateral and adventitious root formation, ethylene is crucial. It interacts with auxin, which boosts ethylene synthesis, and ethylene amplifies auxin signaling, together promoting lateral root development [55]. Auxin and ethylene exert a synergistic effect in governing the growth of primary roots and root hairs, while they function in an antagonistic manner during the formation of lateral roots [56]. Ethylene regulates root hair growth too. ROOT HAIR DEFECTIVE 6-LIKE 4 (RSL4) participates in ethylene-facilitated root hair growth. The ethylene-activated EIN3 transcription factor physically associates with ROOT HAIR DEFECTIVE 6 (RHD6), a well-known positive regulator of hair cells. Additionally, these two factors directly co-activate RSL4 to drive root hair elongation [57]. Rice OsHK1/MHZ1 functions in conjunction with the ethylene receptor, yet exhibits some degree of autonomy from OsEIN2 in its ability to suppress root growth [58].

Overall, ethylene precisely regulates various aspects of root growth through complex signaling pathways, enabling the root system to adapt to environmental changes and ensuring the growth and survival of plants.

### 3.3. Fruit Ripening

During the maturation of succulent fruits, significant alterations in fruit occur, including shifts in color due to the breakdown of chlorophyll or the accumulation of pigments, as well as the release of ethylene [59]. Ethylene plays a crucial role in the maturation of climacteric fruit. During the transition period of fruit ripening, ethylene synthesis increases rapidly and respiration increases [60]. The ethylene signal activates at the beginning of fruit ripening, which induces the expression of *MdEIL1* and its protein stability. Subsequently, *MdEIL1* interacts with the promoter region of *MdMYB1* and stimulates its transcription, making for increased anthocyanin levels and improved fruit pigmentation [61]. In rice, lines of overexpressing *OsETR2* exhibit delayed maturation compared to the control and RNAi lines, and the *osein2/mhz7* mutant also matures later [62]. These studies show that ethylene signaling may affect the flowering of Arabidopsis and rice.

A specific tomato gene known as *SlAP2a*, belonging to the *APETALA2/ERF* family, has been identified as a suppressor of fruit ripening. Inhibition of *SlAP2a* through RNA interference leads to increased production of ethylene in fruits, causing accelerated ripening and influencing the accumulation patterns of carotenoids by perturbing the flux within the carotenoid biosynthesis pathway [63]. In recent years, many tomato fruit ripening mutants were found, which provide important biomaterials for clarifying mechanisms of fruit ripening [64,65]. The NEVER RIPE (nr) mutant exhibited phenotypes of incomplete and delayed fruit ripening [66]. The nr mutant fails to reach maturity primarily due to its insensitivity to the plant hormone ethylene [64,67,68]. The RIPENING INHIBITOR (rin) mutant assumes a pivotal role in the investigation of tomato fruit ripening [69]. This is attributed to its complete inhibition of the alterations in physiological traits associated with ripening. RIN may affect ACS2 enzyme activity by post-transcriptional mechanisms [70]. These inhibited alterations encompass the deposition of red pigments, softening of the fruit, generation of volatile compounds, and the elevation of ethylene levels during the respiratory climacteric phase [71]. In the COLORLESS NON-RIPENING (Cnr) mutant, there is a marked decrease in the expression of genes related to ethylene biosynthesis [72]. This leads to the incapability of the fruit to accumulate ethylene in a normal manner, thereby impeding the ripening process.

Ethylene is crucial for maintaining the quality of fruits during ripening, and its biosynthesis is regulated by nitric oxide (NO) [73]. Nitric oxide–ethylene crosstalk during fruit ripening operates through partial inhibition of ethylene biosynthesis. Mature green tomatoes were treated with the NO synthesis inhibitor. It was found that it reduced the release of endogenous ethylene and delayed the breaker stage of the fruits. This is because the activities of ACS and ACO were inhibited and their related genes SlACS2/4 and SlACO1/3 were downregulated. Meanwhile, the expression of calcium-dependent protein kinase and mitogen-activated protein kinase genes *SlCDPK* (Calcium-Dependent Protein Kinase) 1/2 was also delayed or decreased [74]. It indicates that protein phosphorylation is involved in the reduction of ethylene biosynthesis induced by the NO synthesis inhibitor, ultimately leading to the delay of tomato ripening.

During the fruit ripening process, intricate interactions exist among nitric oxide (NO), a hypoxic environment, and ethylene. Typically, NO functions as an inhibitor, effectively retarding fruit ripening and senescence by suppressing the ethylene biosynthesis pathway. However, under specific circumstances, NO may also indirectly promote ethylene production by activating certain metabolic pathways. The hypoxic environment delays fruit ripening and senescence indirectly by inhibiting both fruit respiration and ethylene biosynthesis. Although ethylene controls hyponasty and aerenchyma formation, NO production apparently regulates hypoxic ethylene biosynthesis [75]. Additionally, the destabilization of group VII ethylene response factors, which are involved in the direct O_2_-sensing mechanism, requires NO [76]. Accumulating research indicates that under hypoxic conditions, NO treatment can further reduce ethylene production. This effect is predominantly achieved through downregulating the transcriptional accumulation of ACS genes.

### 3.4. Leaf Senescence

The most obvious symptom of leaf senescence is chlorosis caused by chlorophyll degradation. The breakdown of chlorophyll may be initiated by endogenous signals and environmental signals, and ethylene is one of the main inducers. The research revealed that EIN3 positively regulates the transcription of chlorophyll degradation genes by directly interacting with their promoter regions [77]. The rice F-Box protein OsFBK (Oryza sativa F-box with Kelch repeat-containing protein) 12 directly interacts with OsSAMS1 (Oryza sativa SPHASE KINASE-ASSOCIATED PROTEIN1-LIKE PROTEIN) to induce its degradation, resulting in a decrease in SAM and ethylene content, thereby inhibiting seed germination and delaying leaf senescence. OsFBK12 is involved in the 26S proteasome pathway by interacting with OsSAMS1. It also focuses on breaking down the substrate OsSAMS1, leading to alterations in ethylene levels that control leaf aging and grain size [78]. ETHYLENE-INSENSITIVE3 (EIN3) is a key transcription factor in ethylene signaling. EIN3 can directly bind to the promoters of *microRNA164 (miR164)* to increase progressively during leaf aging [79]. The gene *EIN3* is associated with senescence and promotes the process of age-related leaf senescence in Arabidopsis by inhibiting the transcription of *miR164.*

## 4. Plant Biotic and Abiotic Stress Regulation in Ethylene Signaling Pathway

As a hormone, ethylene is also widely involved in the context of plant reactions to abiotic and biotic stresses, like drought, high salinity, high or low temperatures, and nutrient deficiencies [80,81] (Table 1).

### 4.1. High Salt Stress

High salt is a remarkable abiotic stress that destroys plant growth and limits crop yield [107]. It has been indicated that the control of cortical microtubule reorganization is essential for the viability of plant cells when exposed to elevated salt levels. Ethylene signaling regulates microtubule reorganization by upregulating the expression of microtubule-stabilizing protein gene *WDL* (Microtubule-binding protein WVD2-like) *5* in reaction to elevated salt stress [88]. Otherwise, the induction of ethylene production is a metabolic response of plants to high salt [108]. The research revealed that in the presence of salt stress, the activity of *OsDOF* (DNA-binding with one finger) *15* was suppressed, leading to a reduction in cell division within the root meristem. Consequently, this inhibition impeded the growth of primary roots [90]. An *ERF* gene, designated as *LchERF*, was successfully isolated from *Lycium chinense* for the first time. The transgenic tobacco lines exhibiting overexpression of *LchERF* demonstrated enhanced tolerance to salt stress conditions during both seed germination and vegetative growth stages [89]. To uncover genes activated in response to salt stress in barrel medic (*Medicago truncatula* L.) seedlings, a cDNA library was developed under salt stress conditions. Notably, a putative *AP2/EREBP* transcription factor was identified, which is known to be pivotal in signal transduction and transcriptional regulation. Additionally, aldolase and sucrose synthase were found to be associated with osmolyte synthesis, showing a marked increase in expression levels, and suggesting their crucial involvement in the plant’s response to salt stress [91].

### 4.2. Cold Stress

Ethylene also works in the response to cold stress. Exogenous ethylene can improve cold tolerance and reduce cold damage to some economic fruits such as bananas [109], apples [95], and tomatoes [110,111]. The research has indicated that the low-temperature-induced ethylene response factor *VaERF092* enhances the tolerance of grapes to low-temperature stress by activating *VaWRKY* (Vitis amurensis WRKY transcription factor) 33 [92]. The ethylene-responsive factor *CdERF1* derived from bermudagrass (*Cynodon dactylon*) enhances cold tolerance. *CdERF1* is involved in positively modulating the plant’s response to cold stress through the activation of stress-related genes, such as lipid transfer protein and lipid transfer protein [93]. A significant finding in the study was the identification of an ethylene response factor *SlERF.B8*, which plays a pivotal role in regulating JA (Jasmonic Acid) biosynthesis in tomato plants subjected to cold stress conditions. The expression of *SlERF.B8* was notably increased in response to both JA treatment and exposure to cold stress. Furthermore, the suppression of *SlERF.B8* resulted in reduced levels of JA accumulation and compromised cold tolerance in tomato plants [94]. In apple seedlings, cold stress can quickly activate the production of ethylene and stimulate the activity of the *MdERF1B* gene. This gene encodes an activator of ethylene signals, which greatly promotes *MdERF1B*-mediated cold tolerance through synergistic action with Malus domestica Cyanidin Synthesis-related basic Helix-Loop-Helix transcription factor 1 *(MdCIbHLH1)*. This process not only reflects the rapid response ability of plants to cold environment, but also demonstrates the key role of the complex regulatory network inside plants in adapting to environmental stress [95].

### 4.3. Drought Stress

To adapt to soil water levels, plants regulate their tissue-specific processes, constantly changing cellular signals within the body, which can lead to premature flowering or stunted development, thereby causing reduced yields. ET is an important hormone regulating drought response [81,112]. The increased expression of *JERF1* in transgenic rice plants results in a notable improvement in their ability to withstand drought conditions. This overexpression leads to the upregulation of stress-responsive genes and heightened production of the osmolyte proline through the regulation of *OsP5CS (Oryza sativa Pyrroline-5-Carboxylate Synthetase)* expression, which encodes the crucial enzyme deltal-pyrroline-5-carboxylate synthetase involved in proline biosynthesis [84]. ERF transcription factors are essential components in the plant stress response. In the present investigation, the molecular mechanism of drought stress response in walnuts was elucidated by identifying a specific ERF transcription factor, *JrERF2-2*, from J. regia. The study clarified that *JrERF2-2* has the capability to enhance plant drought tolerance by modulating the expression of *GSTs* through interaction with *JrWRKY7* [85].

OsARD1 (Oryza sativa Arginine Deiminase 1) is a metal enzyme that facilitates the production of ethylene through biosynthesis in rice by producing methionine, thereby upregulating the activation of drought-related genes and improving rice resilience to drought conditions [86]. In addition, the studies have found that overexpression of *OsEIL2* can induce the expression of the Polygalacturonase gene and reduce pectin content, thereby increasing the sensitivity to drought [87]. Overexpression of *OsEIL2* can increase the sensitivity to drought. The *OsARD1* gene upregulates the transcription of genes associated with drought response, thereby augmenting rice’s resilience to drought stress.

### 4.4. Heat Stress

Exposure of plants to elevated temperatures, known as heat stress (HS), has been shown to have detrimental effects on crop yield and quality. Recent studies have revealed that tomato pollen grains possess the ability to produce ethylene, exhibiting distinct elements of both the ethylene-biosynthesis and -signaling pathways, and are influenced by high-temperature conditions. Pollen grains express specific components of the ethylene signaling pathway, along with various ethylene-responsive factors, among which *SlETR3* (ethylene receptor, also known as NR or never ripe) and *SlCTR2* (constitutive triple response2) have been identified as responsive to heat stress [82]. Moreover, the signaling pathway mediated by ethylene enhances tolerance to high temperatures and controls the transcription levels of heat shock factors in rice seedlings subjected to heat stress. This study shows that, compared with rice seedlings that only experience heat stress, the expression level of heat shock factors such as *HSFA1a* in rice seedlings is increased under the treatment of heat stress and ethylene precursor. Under these conditions, genes related to ethylene signal transduction, such as *EIN2* and *EIL1/2,* are also expressed at higher levels [83].

Hot water treatment of postharvest Mei fruit delays ripening and ethylene production [113] In addition, the results of RT-PCR analysis show that hot water treatment significantly inhibits the expression of ACS, ACO, and ethylene receptor (ETR1 and ERS1) genes in strawberry fruits during storage, but has no significant effect on the expression of the EIN1 gene [114]. The above results indicate that the delay of strawberry fruit senescence by hot water treatment may be related to its inhibition of ethylene synthesis and action. Moreover, hot water treatment and high-temperature ethylene treatment can synergistically promote the antioxidant effect of mature green tomatoes [115].

### 4.5. Nutritional Stress

Ethylene is crucial for plants to respond to nutritional stresses [116]. At the molecular level, it enhances iron absorption by upregulating genes like *AtIRT1 (Arabidopsis thaliana Iron-Regulated Transporter 1)* and *AtFRO2 (Arabidopsis thaliana Ferric Reductase Oxidase 2)* under iron deficiency [96]. Ethylene stabilizes *AtFIT (Arabidopsis thaliana Fer-like Iron Deficiency-Induced Transcription Factor)* protein through the *AtEIN3/AtEIL1* pathway to maintain iron homeostasis [97,117].

In response to phosphorus deficiency, ethylene signaling promotes root hair development. It upregulates *EIN3/EIL1*, which increases *PHT1* expression, resulting in longer and denser root hairs for improved phosphorus uptake [57]. Ethylene also regulates gene expression in leaves to optimize phosphorus use. *GmETO1* in soybeans, for example, enhances root hair growth and low-phosphorus stress resistance by modulating ethylene synthesis and gene expression [99].

Ethylene signaling interacts with nitrogen pathways, such as *EIN2* with *NRT* (Nitrate Transporter) *1.1*, to regulate nitrogen uptake and assimilation. It downregulates *NRT2.1* to balance nitrate absorption and may affect other nitrogen metabolism enzymes or transporters [100]. Nitrogen availability regulates ethylene-related gene expression, indicating a feedback mechanism [118].

Taken together, a schematic review illustrates the complex network of ethylene-mediated responses to different nutritional stresses, highlighting the key genes and pathways involved.

### 4.6. Biotic Stress

Moreover, ethylene plays a crucial role as a hormone in mediating plant–pathogen interactions. Studies have found that ethylene has a positive regulatory role in plant resistance to bacterial pathogens [119]. Various *ERFs* play a role in controlling the ripening of fruits and their response to pathogens, in both climacteric and non-climacteric fruits. These *ERFs* can act in conjunction with or separately from other transcription factors (TFs). The research has shown that numerous *AP2/ERF* transcription factors are implicated in the tomato plant’s reaction to the tomato yellow leaf curl virus, with the *SlERF* protein demonstrating a specific interaction with the GCC-Box [103]. The Arabidopsis ethylene response factor ERF96 is involved in upregulating the expression of defense genes, such as PDF (Plant Defensin) 1.2a, PR (Pathogenesis-Related Protein)-3, and PR-4, through direct interaction with the GCC-box element in their promoters. This action of ERF96 contributes to enhancing plant resistance against necrotic pathogens [101]. The *cytokinin response factor 5 (CRF5*) is a member of the plant-specific *APETALA2* (AP2) ethylene-responsive element-binding proteins (*EREBPs*) family. *CRF5* potentially plays a role in disease resistance by acting as a transcription activator, thereby establishing a functional connection between plant pathogen response and cytokinin signaling [102]. The gene *Cacl-6468* plays a role in regulating resistance in pepper plants against *M. incognita* by influencing the ethylene signaling pathway. Downregulation of Cacl-6468 expression can enhance susceptibility and reduce resistance in pepper cultivar *HDA149* [104].

Furthermore, ethylene is a significant factor in enhancing plant resistance against pests. The activation of plant defense mechanisms in response to insect feeding is controlled through various signaling pathways. Among them, ethylene signaling has been found to heighten the vulnerability of Arabidopsis plants to the generalist herbivore Egyptian cotton worm (*Spodoptera littoralis*). Similarly, the EIN2 mutant, which affects a key element in the ethylene signaling pathway, has been observed to result in increased resistance to Egyptian cotton worms, comparable in effectiveness to the hookless1 mutation [105]. Furthermore, the brown planthopper (BPH) is recognized as a significant pest that specifically impacts rice cultivation, leading to substantial reductions in rice yield. Research has highlighted the crucial role of the OsEBF1 gene, associated with the ET signaling pathway, in enhancing rice’s resistance to BPH. Another key element in this context is OsEBF2, an F-box protein that positively influences rice’s capacity to withstand BPH, presenting a promising target gene for breeding initiatives aimed at bolstering BPH resistance in rice. Therefore, these studies indicate that the ethylene signaling pathway plays a crucial regulatory role in rice’s ability to resist BPH [106].

## 5. Epigenetic Modifications in Ethylene Signaling

**Figure 3:**
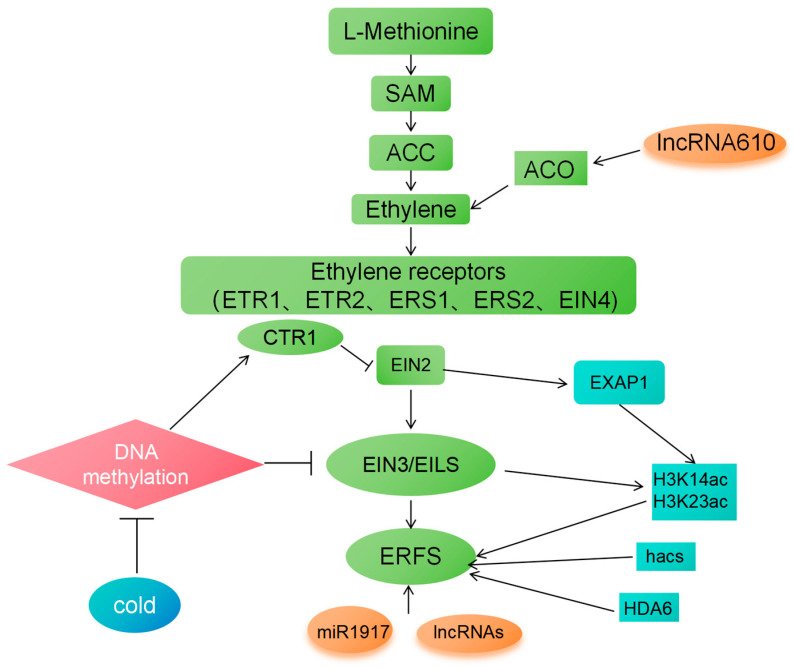
Overview of crosstalk between ethylene and epigenetic regulation described and discussed in this review.

Epigenetic modification refers to the regulation of gene expression rather than altering DNA sequence [120]. This modification can affect multiple levels of gene transcription: splicing, stability, translation, nucleosome assembly, and chromatin structure, thus affecting the physiological and pathological processes of the cells, as well as the phenotype of the offspring [121,122]. Here is an illustration regarding the interaction between ethylene and epigenetic regulation reviewed in this paper (Figure 3).

### 5.1. DNA Methylation and Ethylene Signal Transduction

As the most common form of epigenetic modification, DNA methylation plays a vital regulatory role in most stages of plant growth and development [123]. It can regulate the role of ethylene by inhibiting or activating the expression of genes related to ethylene synthesis and signal transduction. The research has shown that environmental factors, such as stress conditions, can induce changes in DNA methylation status [124]. Hypermethylation in the promoter region of a specific gene may lead to a decrease in gene expression, thus affecting ethylene signal transduction. If the promoter region of a gene related to plant growth and development is methylated, the transcription factor responsible for initiating its expression cannot bind effectively, resulting in abnormal gene expression and affecting the plant’s growth and development process. According to relevant research, under low-temperature stress, different degrees of methylation changes occur in the promoter regions of multiple ethylene signal transduction-related genes in tomatoes. In the present results, the DNA methylation level of the CpG island of *SlEIN3*, *SlERF-A1,* and *SlERT10* increased, and the DNA methylation level of the CpG island of *SlCTR1* decreased in tomato fruit [32]. Meanwhile, the expression levels of *SlEIN3*, *SlERF-A1,* and SlERT10 decreased, while the expression level of *SlCTR1* increased in tomato fruit stored at the low temperature. Moreover, it was observed that the ripening process of tomato fruits was significantly inhibited, indicating that low temperature inhibits ethylene signal transduction and delays the aging of tomato fruits via changing DNA methylation and gene expression. This further verifies the mechanism that DNA methylation regulates plant growth and development by affecting the expression of ethylene signal transduction-related genes. This regulatory mechanism provides an effective molecular mechanism for plants to finely regulate ethylene signal transduction to adapt to environmental changes in a complex environment.

Moreover, DNA methylation can interact with other epigenetic mechanisms to jointly regulate ethylene signal transduction. For example, DNA methylation can affect the expression of histone modifications, thus synergistically regulating the expression of ethylene-related genes. In tomatoes, when a H3K4 demethylase SlJMJ7 loses its function, it leads to an increase in the level of H3K4me3, which directly activates genes related to ethylene biosynthesis and causes DNA hypomethylation mediated by DML2 in fruits, indirectly promoting the expression of ripening-related genes, and jointly resulting in accelerated fruit ripening in the sljmj7 mutant [125]. The results indicate that there is a synergistic effect among epigenetic modifications, which jointly regulate the expression level of ethylene-related genes.

### 5.2. Histone Modification and Ethylene Signal Transduction

Similar to DNA methylation, histone modification is also an important epigenetic modification method. Histones include four types, H2A, H2B, H3, and H4, and their variants [126]. Histone modification regulates gene expression by changing their state. Histone modification refers to post-translational modifications (PTMs) that occur on histones, such as acetylation, methylation, and phosphorylation, which can change the structure and activity of chromatin, thereby affecting the transcription of ethylene signal transduction-related genes [127]. Among them, histone acetylation is usually associated with gene activation, while the effect of histone methylation varies depending on the methylation site and degree. When the receptor receives the ethylene signal, EIN2 located on the endoplasmic reticulum membrane undergoes cleavage. The C-terminal of EIN2 (EIN2-C) is transported into the nucleus, where it promotes an increase in the levels of histone acetylation H3K14Ac and H3K23Ac through a series of epigenetic regulatory mechanisms, thereby regulating the expression of downstream genes. Meanwhile, the regulation of these ethylene-mediated acetylation levels depends on EIN2. In addition, the ethylene signal can also regulate the expression of downstream genes through the core transcriptional activator EIN3, and EIN3 is involved in the regulation of histone acetylation mediated by EIN2 [128]. This mechanism affects the transcription level of ethylene signal transduction-related genes by changing the structure and activity of chromatin, thus finely regulating ethylene signal transduction. Histone acetyltransferases and deacetylases can regulate the expression of ethylene synthesis and signal transduction-related genes, thereby affecting the plant’s response to ethylene. It was found that the mutant involved with hac1 (histone acetyltransferase) exhibits pleiotropic phenotypes, especially being hypersensitive to ethylene in the dark and light, and the transcription level of ethylene-responsive genes in the hac1hac5 double mutant is significantly higher than that in wild-type plants [129,130]. This indicates that histone acetyltransferases play an important role in regulating the expression of ethylene-related genes and the plant’s response to ethylene. Histone modification can also interact with transcription factors to jointly regulate ethylene signal transduction. Transcription factors can bind to specific DNA sequences and then recruit histone-modifying enzymes. After binding to specific DNA sequences, transcription factors bind to histone-modifying enzymes through specific domains or interaction modes. Then, these enzymes modify histones, for example, by adding or removing acetyl, methyl, and other chemical groups on histones, changing the charge and spatial structure of histones, thereby affecting gene expression. In line with this, MaHDA6 is a histone deacetylase in banana (*Musa acuminata*) that targets the promoter of the ethylene-related gene MaERF11/15, and this gene may be involved in regulating fruit ripening [131].

### 5.3. Non-Coding RNAs and Ethylene Signal Transduction

Non-coding RNAs play an important and diverse role in gene expression regulation. Although they do not encode proteins, they play a key role in many aspects, such as plant growth, development, and environmental adaptation, mainly including micro RNA (miRNA) and long no-coding RNA (lncRNA) [132]. In tomatoes, studies on genes related to the ethylene signal pathway have found the synergistic effect of multiple epigenetic regulatory mechanisms. For example, wild-type and LeERF1 transgenic tomato fruits were studied, and a regulatory network of small RNAs and genes related to the ethylene signal pathway was found [133], including the interaction relationship between lncRNAs and miRNAs, where two lncRNAs are the precursors of three miRNAs, and four lncRNAs can be preceded by five miRNAs. MiRNAs can bind to the mRNAs of key genes in the ethylene signaling pathway, inhibit its translation or promote its degradation, thus affecting the transmission of ethylene signals. For example, In Arabidopsis, some miRNAs can target ethylene receptor genes or downstream signal transduction components to regulate ethylene responses [134]. *MicroRNA1917* targets CTR4 splice variants to regulate ethylene responses in tomato [134]. In *Sly-miR1917*-overexpressing plants, enhanced ethylene signaling is accompanied by upregulation of ethylene biosynthesis and signaling genes, and increases ethylene emission. LncRNA can participate in the regulation of ethylene signal transduction in multiple ways. It can act as a decoy molecule to bind transcription factors or other regulatory proteins, thus affecting the expression of ethylene-related genes. In apple, *MdLNC610* participates in the regulation of high light-induced anthocyanin production by functioning as a positive regulator to promote *MdACO1* expression and ethylene biosynthesis at a specific stage of apple fruit development [135]. The team further studied and identified 397 *lncRNAs* in overexpressing and suppressing *LeERF1* transgenic and control tomato fruits, among which 12 *lncRNAs* are differentially expressed in transgenic and control fruits, and many target genes of lncRNAs are related to ethylene signals, such as auxin response factors, F-box proteins, *ERFs*, and *MADS*-box proteins [133]. These research results indicate that lncRNA plays an important role in the ethylene signal transduction process. These non-coding RNAs together form a complex regulatory network. In-depth study of them will help us better understand the mechanism of plant life activities.

Taken together, DNA methylation, histone modification, and non-coding RNAs play significant roles in ethylene signal transduction. Future research should focus on exploring the comprehensive effects of these epigenetic modifications and their potential applications in plant biotechnology and agriculture.

## 6. Correlation Between Ethylene and Respiration

**Figure 4:**
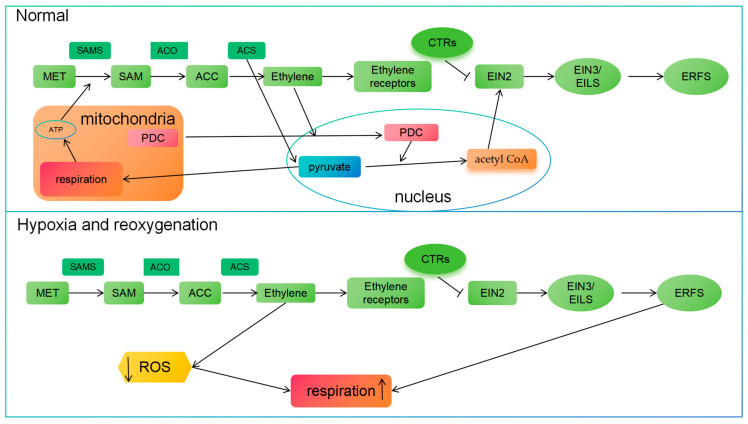
Correlation between ethylene and respiration described and discussed in this review.

Ethylene is pivotal in regulating plant development and controlling respiration. Respiration is the cornerstone of energy metabolism in plant cells, encompassing the electron transport chain within mitochondria. The ripening of climacteric fruits is marked by a substantial rise in ethylene production and a concomitant increase in the intensity of respiration [60]. Moreover, the relationship between ethylene and respiration is depicted and discussed in Figure 4.

The involvement of ethylene in the process of fruit development and ripening is closely linked to respiration, which impacts the rate and quality of fruit ripening through the regulation of ethylene production and signaling pathways [136,137]. Recent research findings indicate a direct link between ethylene signaling and cellular metabolism. In the presence of ethylene, the functional pyruvate dehydrogenase complex (PDC) can translocate from mitochondria to the nucleus. In the nuclear pool, it converts pyruvate into acetyl CoA. This acetyl CoA is utilized for the EIN2-directed acetylation of core histones. Specifically, it supplies acetyl CoA to increase histone acetylation at H3K14 and H3K23, thereby regulating transcriptional regulation in ethylene responses. Furthermore, since respiration is the cornerstone of energy metabolism in plant cells, it is speculated that ethylene may subsequently affect plant respiration. Studies have revealed that, apart from facilitating the conversion of SAM to ACC, ACS proteins in angiosperms typically exhibit Cβ-S lyase (cysteine-S-conjugate β-lyase) activity, which mediates the conversion of cystine or cysteine into pyruvate. Pyruvate serves as the final product of glycolysis and acts as the energy source for the mitochondrial tricarboxylic acid cycle [24]. Therefore, in addition to promoting ethylene synthesis, ACS2 and ACS4, which are highly expressed during fruit ripening, may also provide substrates for mitochondrial aerobic respiration, indicating that ethylene and mitochondria interact during fruit ripening. In addition, ethylene can regulate the activity of respiratory enzymes such as cytochrome oxidase and alternative oxidase, thereby altering the efficiency of respiratory electron transfer and energy production [25,26].

Ethylene can regulate the expression of genes related to mitochondrial biosynthesis and function, promoting the recovery of mitochondrial activity during reoxygenation [138]. Ethylene signaling and respiration work synergistically during critical stages of plant growth, such as seed germination and fruit ripening. During seed germination, ethylene can promote an increase in respiration rate, providing necessary energy for the growth and development of seedlings [51,139]. Meanwhile, respiration can provide the intermediates and energy required for ethylene biosynthesis, thereby forming a positive feedback loop. During fruit ripening, ethylene can induce upregulation of respiratory enzymes and activation of respiratory electron transport chains, leading to an increase in respiratory rate and accumulation of energy and metabolites required for fruit ripening [140].

In addition, ethylene signaling significantly controls energy and reactive oxygen species (ROS) metabolism during hypoxia and reoxygenation processes. Ethylene is quickly sequestered in submersed plant cells, enhancing hypoxia acclimation [141]. Breathing is crucial in plants’ response to hypoxia and subsequent reoxygenation. During periods of hypoxia, plants need to adjust their energy metabolism to cope with limited oxygen supply. They can suppress respiration to reduce oxygen consumption, and, at the same time, they can activate alternative pathways such as anaerobic respiration to generate some energy [93]. When plants are reoxygenated, they need to quickly restore respiration to meet the increased energy required for recovery and growth. This has a significant impact on metabolic remodeling in hypoxia and reoxygenation responses by stimulating and controlling the expression of plant ERFVIII transcription factors genes related to hypoxia adaptation, autophagy, and reactive oxygen species detoxification [138]. For example, ethylene triggers the activation of ERFVIII transcription factors *SNORKEL1* and *2* in rice, leading to increased internode elongation, which is a mechanism for escaping hypoxic conditions by restoring gas exchange [140]. On the contrary, ethylene also induces ERFVIII subunit 1A (*SUB1A*). In rice, certain ERFVIII homologs are believed to be isolated from the PRT6 N-de duplication pathway, leading to their protein levels being directly regulated by ethylene signaling [142]. This study indicates that ethylene promotes the removal of ROS during the reoxygenation process in Arabidopsis [99]. EIN3 and its target transcription factors play a direct regulatory role in the expression of many genes encoding proteins associated with increased levels of ROS in various plant species. These genes cover key coding sequences responsible for the biosynthesis of carbonic anhydrase, catalase, peroxidase, and ascorbic acid, which together constitute important mechanisms for plants to cope with environmental stress and promote growth and development [143,144,145,146].

In summary, ethylene signaling is integral to respiration and plays a key role in plant growth, development, and environmental adaptation. The intricate relationship between ethylene and respiration warrants further investigation.

## 7. Conclusions and Prospects

Ethylene currently stands as the singular plant gas hormone identified. It serves a crucial function in several aspects of plant growth and development, holding notable importance in the realm of agricultural production. Additionally, the role of epigenetic modifications and the correlation with respiration have been explored.

However, there are still gaps in our understanding. Regarding signaling pathway interactions, while the core pathway is known, the full extent of its interactions with other signaling pathways, especially in complex stress situations, remains unclear [13]. We are unaware of all the potential crosstalk mechanisms and how they might be influenced by diverse environmental conditions. Understanding these interactions is crucial for predicting plant responses accurately in real-world scenarios and for developing more effective strategies to improve plant stress tolerance and growth. In terms of species comparison, the research has predominantly focused on a few model plants, and there is a deficiency of comprehensive cross-species comparisons [35]. We are lacking knowledge of the complete range of variability and universality of ethylene signaling among different plant species. This knowledge is crucial for applying the results from model plants to a broader range of agriculturally important species and for comprehending the evolution and adaptation of ethylene signaling mechanisms. For molecular mechanisms in certain areas, such as epigenetic modifications and respiration regulation, detailed molecular mechanisms are not thoroughly understood. For instance, the precise interactions of non-coding RNAs with other components in the ethylene signaling pathway, along with the cell metabolism and molecular mechanisms of ethylene’s effect on respiration, require further elucidation. A more profound understanding of these molecular mechanisms is requisite for targeted genetic engineering and manipulation of ethylene signaling, which may result in more effective plant improvement strategies.

In the text, we describe numerous examples about ethylene signal transduction in plant growth, development, and the processes of responding to various stresses. The main function of these examples is to intuitively and effectively illustrate the crucial role that ethylene signal transduction plays in the corresponding physiological processes, rather than being a comprehensive review of all the phenomena in this field. In fact, throughout the entire life cycle of plants and their adaptation to the complex ecological environment, the specific manifestations and action mechanisms of ethylene signal transduction are extremely diverse and complex. There are still a large number of research achievements and examples that have not been mentioned and need to be further explored and integrated.

Future research ought to concentrate on multiple directions. Firstly, it should unveil the complete spectrum of crosstalk between ethylene signaling and other pathways. This may entail studying plants under diverse stress combinations and employing advanced molecular techniques to identify novel interaction points and regulatory mechanisms. Secondly, more extensive cross-species research is requisite to compare ethylene signaling mechanisms. This might involve studying a wide variety of plant species from different families and habitats to comprehend the commonalities and differences in the functioning of ethylene signaling. Finally, in-depth investigations on the molecular mechanisms of epigenetic modifications and respiration regulation in relation to ethylene signaling are crucial. This could involve detailed biochemical and genetic analyses to ascertain the exact roles and interactions of various components, which will assist in formulating more precise genetic modification strategies for enhancing plant growth and stress tolerance.

Moreover, the role of ethylene in distinct plant species and different growth stages may vary, which necessitates more cross-species and cross-stage comparative studies for elucidation. Simultaneously, with the advancement of science and technology and the intensification of research, some new pathways independent of the core signal transduction elements and the new role of the core signal transduction elements in the classical ethylene signaling pathway have been detected. Whether there are new ethylene signaling pathways and new regulatory factors, as well as the relationships between these potential pathways and classical signaling pathways, require further exploration.

## Figures and Tables

**Table 1:** The effects of ethylene signaling on biotic and abiotic stresses in plants.

Biotic/Abiotic Stress	Action	Plant Species	Reference
Heat stress	The signaling of ethylene in tomato pollen grains exhibits sensitivity to heat stress.	*Solanum lycopersicum*	[82]
	Increased levels of EIN2 expression were observed in rice seedlings subjected to heat stress.	*Oryza sativa*	[83]
Drought stress	The increased expression of *ERF1* resulted in a notable improvement in the drought resistance of genetically modified rice plants.	*Oryza sativa*	[84]
	*JrERF2-2* can enhance plant resistance to drought stress by interacting with *JrWRKY7* to regulate the expression of *GSTs*.	*Juglans regia*	[85]
	The *OsARD1* upregulates the expression of genes associated with drought response, thereby improving rice’s ability to withstand drought conditions.	*Oryza sativa*	[86]
	Overexpression of *OsEIL2* can increase the sensitivity to drought.	*Oryza sativa*	[87]
Salt stress	The reassembly of microtubules is regulated by ethylene signaling through the upregulation of *WDL5* expression in response to salt stress.	*Arabidopsis thaliana*	[88]
	The enhanced expression of *LchERF* resulted in increased tolerance to salt stress.	*Lycium chinense*	[89]
	The expression of *OsDOF15* was inhibited under salt stress.	*Oryza sativa*	[90]
	Identification of genes that are activated in response to salt stress in seedlings of *Medicago truncatula* L.	*Medicago truncatula*	[91]
Cold stress	*VaERF092* controls the activity of the transcription factor *VaWRKY33,* enhancing resistance to cold stress.	*Vitis amurensis*	[92]
	*CdERF1* in bermudagrass plays a role in enhancing cold tolerance.	*Cynodon dactylon*	[93]
	The *SlERF.B8* protein induces the biosynthesis of JA to enhance cold tolerance in tomato plants.	*Solanum lycopersicum*	[94]
	Ethylene enhances the cold resistance of apples through the regulatory module *MdERF1B-MdCIbHLH1*.	*Malus domestica*	[95]
Nutrition stress	Ethylene participates in the upregulation of several Fe acquisition genes of Arabidopsis, such as *AtFIT*, *AtFRO2*, and *AtIRT1.*	*Arabidopsis thaliana*	[96]
	*AtEIN3* is a nuclear protein gene that functions immediately after *AtERF1* in ethylene signaling.	*Arabidopsis thaliana*	[97]
	*EIN3/EIL1* activates *PHT1* to enhance the absorption of phosphorus.	*Arabidopsis thaliana*	[98]
	*GmETO1* enhances the tolerance of soybeans to low phosphorus stress.	*Glycine max*	[99]
	Ethylene, in turn, downregulates the expression of *NRT2.1* and reduces the high-affinity absorption of nitrate.	*Arabidopsis thaliana*	[100]
Biotic stress	*SlERFs* are involved in the response of tomato yellow leaf curl virus	*Solanum lycopersicum*	[101]
	Increased expression of *CRF5* enhances resistance to pathogens in Arabidopsis plants.	*Arabidopsis thaliana*	[102]
Biotic stress	*AP2/ERF* transcription factors play a role in the tomato yellow leaf curl virus response.	*Solanum lycopersicum*	[103]
	*ERF96* positively regulates Arabidopsis resistance to necrotrophic pathogens.	*Arabidopsis thaliana*	[101]
	The study focuses on Pepper ethylene-responsive proteinase inhibitor *Cacl-6468* and its impact on enhancing resistance against Meloidogyne incognita.	*Capsicum annuum*	[104]
	*Ein2* enhances resistance to Egyptian cotton worms.	*Gossypium hirsutum*	[105]
	The ethylene signaling pathway exerts a negative regulatory effect on the resistance of rice plants to brown planthoppers.	*Oryza sativa*	[106]

## Data Availability

Not applicable.

## Abbreviations

**Abbreviation**	**Definition**
ACC	1-Aminocyclopropane-1-caroboxylic acid
ACO	1-Aminocyclopropane-1-caroboxylic acid oxidase
ACS	1-Aminocyclopropane-1-caroboxylic acid synthase
AOX	Alternative Oxidase
AtFIT	Arabidopsis thaliana Fer-like Iron Deficiency-Induced Transcription Factor
AtFRO2	Arabidopsis thaliana Ferric Reductase Oxidase 2
AtIRT1	Arabidopsis thaliana Iron-Regulated Transporter1
BPH	brown planthopper
CDPK	Calcium-Dependent Protein Kinase
CNR	Colorless Non-Ripening
COX	Cytochrome Oxidase
EBF	EIN3-Binding F-BOX Protein
ER	endoplasmic reticulum
ERF	Ethylene Response Factors
ERS	RESPONSE SENSOR
ETR	EIN2-Targeting Protein
EXP	Expansin Genes
GST	Glutathione S-Transferase
hac	histone acetyltransferase
JA	Jasmonic Acid
lncRNA	long non-coding RNA
LTP	Lipid transfer protein
MdCIbHLH	Malus domestica Cyanidin Synthesis-related basic Helix-Loop-Helix transcription factor
MHZ1/OsHK1	Multi-Hit Zinc-Finger Protein 1/ Oryza sativa High-Affinity Potassium Transporter 1
miRNA	micro RNA
MYB	v-myeloblastosis viral oncogene homolog factor
NO	nitric oxide
NR	Never Ripe
NRT	Nitrate Transporter
OsARD1	Oryza sativa Arginine Deiminase 1
OsFBK12	Oryza sativa F-box with Kelch repeat-containing protein 12
OsP5CS	Oryza sativa Pyrroline-5-Carboxylate Synthetase
OsSAMS1	Oryza sativa Sphase Kinase-Associated protein1-Like protein
PDC	pyruvate dehydrogenase complex
PDF	Plant Defensin 1.2a
PR	Pathogenesis-Related Protein
PTMs	post-translational modifications
RHD	Root Hair Defective
RIN	RIPENING INHIBITOR
ROS	reactive oxygen species
RSL	Root Hair Defective 6-LIKE
SAM	S-adenosyl-l-methionine
slAP2a	Solanum lycopersicum APETALA2a
SUB1A	subunit 1A
VaWRKY	Vitis amurensis WRKY transcription factor
WDL5	Microtubule-binding protein WVD2-like 5

## References

[1] Zhang Z.G., Zhou H.L., Chen T., Gong Y., Cao W.H., Wang Y.J., Zhang J.S., Chen S.Y. (2004). Evidence for serine/threonine and histidine kinase activity in the tobacco ethylene receptor protein NTHK2. Plant Physiol..

[2] Huang W., Kang Y. (2013). Research Advances in Ethylene Regulation to Growth and Development of Seedling Root. Chin. Agric. Sci. Bull..

[3] Morgan P.W., Drew M.C. (1997). Ethylene and plant responses to stress. Physiol. Plant..

[4] Cao Y.-R., Chen S.-Y., Zhang J.-S. (2008). Ethylene signaling regulates salt stress response: An overview. Plant Signal. Behav..

[5] Husain T., Fatima A., Suhel M., Singh S., Sharma A., Prasad S.M., Singh V.P. (2020). A brief appraisal of ethylene signaling under abiotic stress in plants. Plant Signal. Behav..

[6] Yang S.F., Hoffman N.E. (1984). Ethylene Biosynthesis and Its Regulation in Higher-Plants. Annu. Rev. Plant Physiol. Plant Mol. Biol..

[7] Yang C., Lu X., Ma B., Chen S.Y., Zhang J.S. (2015). Ethylene signaling in rice and Arabidopsis: Conserved and diverged aspects. Mol. Plant.

[8] Larsen P.B. (2015). Mechanisms of ethylene biosynthesis and response in plants. Essays Biochem..

[9] Bürstenbinder K., Rzewuski G., Wirtz M., Hell R., Sauter M. (2007). The role of methionine recycling for ethylene synthesis in Arabidopsis. Plant J..

[10] Lyzenga W.J., Stone S.L. (2012). Regulation of ethylene biosynthesis through protein degradation. Plant Signal. Behav..

[11] Alexander L., Grierson D. (2002). Ethylene biosynthesis and action in tomato: A model for climacteric fruit ripening. J. Exp. Bot..

[12] Ju C., Chang C. (2015). Mechanistic Insights in Ethylene Perception and Signal Transduction. Plant Physiol..

[13] Merchante C., Alonso J.M., Stepanova A.N. (2013). Ethylene signaling: Simple ligand, complex regulation. Curr. Opin. Plant Biol..

[14] Binder B.M. (2020). Ethylene signaling in plants. J. Biol. Chem..

[15] Park C., Lee H.Y., Yoon G.M. (2021). The regulation of ACC synthase protein turnover: A rapid route for modulating plant development and stress responses. Curr. Opin. Plant Biol..

[16] De Paepe A., Van Der Straeten D. (2005). Ethylene biosynthesis and signaling: An overview. Vitam. & Horm..

[17] Zhou Y., Xiong Q., Yin C.-C., Ma B., Chen S.-Y., Zhang J.-S. (2020). Ethylene Biosynthesis, Signaling, and Crosstalk with Other Hormones in Rice. Small Methods..

[18] Tucker M.L., Kim J., Wen C.K. (2017). Treatment of Plants with Gaseous Ethylene and Gaseous Inhibitors of Ethylene Action. Methods Mol. Biol..

[19] Zhang Z., Chen Y., Shao Y. (2009). Review of Research Advances on Phytohormones Regulating Plant Defense Responses. Crops.

[20] Lin Z., Zhong S., Grierson D. (2009). Recent advances in ethylene research. J. Exp. Bot..

[21] Squier S.A., Taylor G.E., Selvidge W.J., Gunderson C.A. (1985). Effect of Ethylene and Related Hydrocarbons on Carbon Assimilation and Transpiration in Herbaceous and Woody Species. Environ. Sci. Technol..

[22] Kan J., Zhang Q., Wan B., Liu J., Jin C. (2015). Effect of Exogenous Ethylene on Mitochondrial Cytochrome C Oxidase during Postharvest Storage of Peach Fruit. Food Sci..

[23] Fugate K.K., Suttle J.C., Campbell L.G. (2010). Ethylene production and ethylene effects on respiration rate of postharvest sugarbeet roots. Postharvest Biol. Technol..

[24] Xu C., Hao B.W., Sun G.L., Mei Y.Y., Sun L.F., Sun Y.M., Wang Y.B., Zhang Y.Y., Zhang W., Zhang M.Y. (2021). Dual activities of ACC synthase: Novel clues regarding the molecular evolution of *ACS* genes. Sci. Adv..

[25] Moller I.M., Rasmusson A.G., Siedow J.N., Vanlerberghe G.C. (2010). The product of the alternative oxidase is still H_2_O. Arch. Biochem. Biophys..

[26] Xu F., Yuan S., Zhang D.W., Lv X., Lin H.H. (2012). The role of alternative oxidase in tomato fruit ripening and its regulatory interaction with ethylene. J. Exp. Bot..

[27] Tsai K.J., Chou S.J., Shih M.C. (2014). Ethylene plays an essential role in the recovery of Arabidopsis during post-anaerobiosis reoxygenation. Plant Cell Environ..

[28] Yang L., Li N., Liu Y., Miao P.F., Liu J., Wang Z. (2023). Updates and Prospects: Morphological, Physiological, and Molecular Regulation in Crop Response to Waterlogging Stress. Agronomy.

[29] Hsu F.C., Chou M.Y., Peng H.P., Chou S.J., Shih M.C. (2011). Insights into hypoxic systemic responses based on analyses of transcriptional regulation in Arabidopsis. PLoS ONE.

[30] Li X., Lin C., Lan C., Tao Z. (2024). Genetic and epigenetic basis of phytohormonal control of floral transition in plants. J. Exp. Bot..

[31] Talarico E., Zambelli A., Araniti F., Greco E., Chiappetta A., Bruno L. (2024). Unravelling the Epigenetic Code: DNA Methylation in Plants and Its Role in Stress Response. Epigenomes.

[32] Chen T., Duan W. (2023). DNA methylation changes were involved in inhibiting ethylene signaling and delaying senescence of tomato fruit under low temperature. Biologia.

[33] Yang S.F., Yip W.K., Satoh S., Miyazaki J.H., Jiao X., Liu Y., Su L.Y., Peiser G.D. (1988). Metabolic aspects of ethylene biosynthesis. Plant Growth Subst..

[34] Fluhr R., Mattoo A.K. (1996). Ethylene-Biosynthesis and perception. Crit. Rev. Plant Sci..

[35] Zhao H., Yin C.C., Ma B., Chen S.Y., Zhang J.S. (2021). Ethylene signaling in rice and Arabidopsis: New regulators and mechanisms. J. Integr. Plant Biol..

[36] Sakai H., Hua J., Chen Q.G., Chang C., Medrano L.J., Bleecker A.B., Meyerowitz E.M. (1998). ETR2 is an ETR1-like gene involved in ethylene signaling in Arabidopsis. Proc. Natl. Acad. Sci. USA.

[37] Jia W., Liu G., Zhang P., Li H., Peng Z., Wang Y., Jemrić T., Fu D. (2023). The Ubiquitin-26S Proteasome Pathway and Its Role in the Ripening of Fleshy Fruits. Int. J. Mol. Sci..

[38] Song J., Zhu C., Zhang X., Wen X., Liu L., Peng J., Guo H., Yi C. (2015). Biochemical and Structural Insights into the Mechanism of DNA Recognition by Arabidopsis ETHYLENE INSENSITIVE3. PLoS ONE.

[39] Fatma M., Asgher M., Iqbal N., Rasheed F., Sehar Z., Sofo A., Khan N.A. (2022). Ethylene Signaling under Stressful Environments: Analyzing Collaborative Knowledge. Plants.

[40] Müller M., Munné-Bosch S. (2015). Ethylene Response Factors: A Key Regulatory Hub in Hormone and Stress Signaling. Plant Physiol..

[41] Wu C., Cheng H., Li S., Zuo D., Lin Z., Zhang Y., Lv L., Wang Q., Song G. (2021). Molecular cloning and characterization of GhERF105, a gene contributing to the regulation of gland formation in upland cotton (*Gossypium hirsutum* L.). BMC Plant Biol..

[42] Lei C., Dang Z., Zhu M., Zhang M., Wang H., Chen Y., Zhang H. (2024). Identification of the ERF gene family of Mangifera indica and the defense response of MiERF4 to *Xanthomonas campestris* pv. mangiferaeindicae. Gene.

[43] Sato H., Köhler C. (2022). Genomic imprinting regulates establishment and release of seed dormancy. Curr. Opin. Plant Biol..

[44] Carruggio F., Onofri A., Catara S., Impelluso C., Castrogiovanni M., Lo Cascio P., Cristaudo A. (2021). Conditional Seed Dormancy Helps *Silene hicesiae* Brullo & Signor. Overcome Stressful Mediterranean Summer Conditions. Plants.

[45] Wang X., Yesbergenova-Cuny Z., Biniek C., Bailly C., El-Maarouf-Bouteau H., Corbineau F. (2018). Revisiting the Role of Ethylene and N-End Rule Pathway on Chilling-Induced Dormancy Release in Arabidopsis Seeds. Int. J. Mol. Sci..

[46] Yao W., Wang L., Zhou B., Wang S., Li R., Jiang T. (2016). Over-expression of poplar transcription factor ERF76 gene confers salt tolerance in transgenic tobacco. J. Plant Physiol..

[47] Li X., Fei R., Chen Z., Fan C., Sun X. (2020). Plant hormonal changes and differential expression profiling reveal seed dormancy removal process in double dormant plant-herbaceous peony. PLoS ONE.

[48] Pirrello J., Jaimes-Miranda F., Sanchez-Ballesta M.T., Tournier B., Khalil-Ahmad Q., Regad F., Latche A., Pech J.C., Bouzayen M. (2006). Sl-ERF2, a tomato ethylene response factor involved in ethylene response and seed germination. Plant Cell Physiol..

[49] Fu J.Y., Pei W.Z., He L.Q., Ma B., Tang C., Zhu L., Wang L.P., Zhong Y.Y., Chen G., Wang Q. (2023). ZmEREB92 plays a negative role in seed germination by regulating ethylene signaling and starch mobilization in maize. PLoS Genet..

[50] Iglesias-Moya J., Cebrián G., Garrido D., Martínez C., Jamilena M. (2023). The ethylene receptor mutation etr2b reveals crosstalk between ethylene and ABA in the control of *Cucurbita pepo* germination. Physiol. Plant..

[51] Qin H., Xiao M.G., Li Y.X., Huang R.F. (2024). Ethylene Modulates Rice Root Plasticity under Abiotic Stresses. Plants.

[52] Qin H., Ma C., Zhou Y., Miao Y., Huang R. (2020). Molecular Modulation of Root Development by Ethylene. Small Methods.

[53] Deng Y., Wang C., Zhang M., Wei L., Liao W. (2022). Identification of Key Genes during Ethylene-Induced Adventitious Root Development in Cucumber (*Cucumis sativus* L.). Int. J. Mol. Sci..

[54] Cho H.T., Cosgrove D.J. (2002). Regulation of root hair initiation and expansin gene expression in Arabidopsis. Plant Cell.

[55] Qin H., Huang R. (2018). Auxin Controlled by Ethylene Steers Root Development. Int. J. Mol. Sci..

[56] Negi S., Ivanchenko M.G., Muday G.K. (2008). Ethylene regulates lateral root formation and auxin transport in *Arabidopsis thaliana*. Plant J..

[57] Feng Y., Xu P., Li B., Li P., Wen X., An F., Gong Y., Xin Y., Zhu Z., Wang Y. (2017). Ethylene promotes root hair growth through coordinated EIN3/EIL1 and RHD6/RSL1 activity in *Arabidopsis*. Proc. Natl. Acad. Sci. USA.

[58] Zhao H., Duan K.-X., Ma B., Yin C.-C., Hu Y., Tao J.-J., Huang Y.-H., Cao W.-Q., Chen H., Yang C. (2020). Histidine kinase MHZ1/OsHK1 interacts with ethylene receptors to regulate root growth in rice. Nat. Commun..

[59] Klee H.J., Giovannoni J.J. (2011). Genetics and Control of Tomato Fruit Ripening and Quality Attributes. Annu. Rev. Genet..

[60] Giovannoni J., El-Rakshy S. Genetic regulation of tomato fruit ripening and development and implementation of associated genomics tools. Proceedings of the 5th International Postharvest Symposium.

[61] An J.-P., Wang X.-F., Li Y.-Y., Song L.-Q., Zhao L.-L., You C.-X., Hao Y.-J. (2018). EIN3-LIKE1, MYB1, and ETHYLENE RESPONSE FACTOR3 Act in a Regulatory Loop That Synergistically Modulates Ethylene Biosynthesis and Anthocyanin Accumulation. Plant Physiol..

[62] Ma B., He S.-J., Duan K.-X., Yin C.-C., Chen H., Yang C., Xiong Q., Song Q.-X., Lu X., Chen H.-W. (2013). Identification of Rice Ethylene-Response Mutants and Characterization of *MHZ7/OsEIN2* in Distinct Ethylene Response and Yield Trait Regulation. Mol. Plant.

[63] Chung M.Y., Vrebalov J., Alba R., Lee J., McQuinn R., Chung J.D., Klein P., Giovannoni J. (2010). A tomato (*Solanum lycopersicum*) *APETALA2/ERF* gene, *SlAP2a*, is a negative regulator of fruit ripening. Plant J..

[64] Liu Y., Tang M., Liu M., Su D., Chen J., Gao Y., Bouzayen M., Li Z. (2020). The Molecular Regulation of Ethylene in Fruit Ripening. Small Methods.

[65] Wang R., Lammers M., Tikunov Y., Bovy A.G., Angenent G.C., de Maagd R.A. (2020). The rin, nor and Cnr spontaneous mutations inhibit tomato fruit ripening in additive and epistatic manners. Plant Sci..

[66] Lanahan M.B., Yen H.C., Giovannoni J.J., Klee H.J. (1994). The Never Ripe Mutation Blocks Ethylene Perception in Tomato. Plant Cell.

[67] Martel C., Vrebalov J., Tafelmeyer P., Giovannoni J.J. (2011). The Tomato MADS-Box Transcription Factor RIPENING INHIBITOR Interacts with Promoters Involved in Numerous Ripening Processes in a COLORLESS NONRIPENING-Dependent Manner. Plant Physiol..

[68] Osorio S., Alba R., Damasceno C.M.B., Lopez-Casado G., Lohse M., Zanor M.I., Tohge T., Usadel B., Rose J.K.C., Fei Z. (2011). Systems Biology of Tomato Fruit Development: Combined Transcript, Protein, and Metabolite Analysis of Tomato Transcription Factor (*nor*, *rin*) and Ethylene Receptor (*Nr*) Mutants Reveals Novel Regulatory Interactions. Plant Physiol..

[69] Li S., Zhu B., Pirrello J., Xu C., Zhang B., Bouzayen M., Chen K., Grierson D. (2020). Roles of RIN and ethylene in tomato fruit ripening and ripening-associated traits. New Phytol..

[70] Ito Y., Nishizawa-Yokoi A., Endo M., Mikami M., Shima Y., Nakamura N., Kotake-Nara E., Kawasaki S., Toki S. (2017). Re-evaluation of the rin mutation and the role of RIN in the induction of tomato ripening. Nat. Plants.

[71] Ito Y., Kitagawa M., Ihashi N., Yabe K., Kimbara J., Yasuda J., Ito H., Inakuma T., Hiroi S., Kasumi T. (2008). DNA-binding specificity, transcriptional activation potential, and the rin mutation effect for the tomato fruit-ripening regulator RIN. Plant J..

[72] Manning K., Tor M., Poole M., Hong Y., Thompson A.J., King G.J., Giovannoni J.J., Seymour G.B. (2006). A naturally occurring epigenetic mutation in a gene encoding an SBP-box transcription factor inhibits tomato fruit ripening. Nat. Genet..

[73] Mukherjee S. (2019). Recent advancements in the mechanism of nitric oxide signaling associated with hydrogen sulfide and melatonin crosstalk during ethylene-induced fruit ripening in plants. Nitric Oxide.

[74] Yang Y., Zheng Y., Liu C., Chen L., Ma J., Sheng J., Shen L. (2016). Inhibition of nitric oxide synthesis delayed mature-green tomato fruits ripening induced by inhibition of ethylene. Sci. Hortic..

[75] Gupta K.J., Mur L.A.J., Wany A., Kumari A., Fernie A.R., Ratcliffe R.G. (2020). The role of nitrite and nitric oxide under low oxygen conditions in plants. New Phytol..

[76] Pucciariello C., Perata P. (2017). New insights into reactive oxygen species and nitric oxide signalling under low oxygen in plants. Plant Cell Environ..

[77] Qiu K., Li Z.P., Yang Z., Chen J.Y., Wu S.X., Zhu X.Y., Gao S., Gao J., Ren G.D., Kuai B.K. (2015). EIN3 and ORE1 Accelerate Degreening during Ethylene-Mediated Leaf Senescence by Directly Activating Chlorophyll Catabolic Genes in *Arabidopsis*. PLoS Genet..

[78] Chen Y., Xu Y.Y., Luo W., Li W.X., Chen N., Zhang D.J., Chong K. (2013). The F-Box Protein OsFBK12 Targets OsSAMS1 for Degradation and Affects Pleiotropic Phenotypes, Including Leaf Senescence, in Rice. Plant Physiol..

[79] Li Z.H., Peng J.Y., Wen X., Guo H.W. (2013). *ETHYLENE-INSENSITIVE3* Is a Senescence-Associated Gene That Accelerates Age-Dependent Leaf Senescence by Directly Repressing *miR164* Transcription in *Arabidopsis*. Plant Cell.

[80] Zhao H., Chen S., Zhang J. (2016). Ethylene Signaling Pathway in Regulating Plant Response to Abiotic Stress. Biotechnol. Bull..

[81] Yu Y.W., Huang R.F. (2013). Ethylene and plant resistance to adversity. J. Agric. Sci. Technol..

[82] Jegadeesan S., Beery A., Altahan L., Meir S., Pressman E., Firon N. (2018). Ethylene production and signaling in tomato (*Solanum lycopersicum*) pollen grains is responsive to heat stress conditions. Plant Reprod..

[83] Wu Y.-S., Yang C.-Y. (2019). Ethylene-mediated signaling confers thermotolerance and regulates transcript levels of heat shock factors in rice seedlings under heat stress. Bot. Stud..

[84] Zhang Z.J., Li F., Li D.J., Zhang H.W., Huang R.F. (2010). Expression of ethylene response factor JERF1 in rice improves tolerance to drought. Planta.

[85] Yang G.Y., Peng S.B., Wang T.Y., Gao X.Q., Li D.P., Li M.G., Chen S.W., Xu Z.G. (2021). Walnut ethylene response factor JrERF2-2 interact with JrWRKY7 to regulate the GSTs in plant drought tolerance. Ecotoxicol. Environ. Saf..

[86] Liang S.S., Xiong W., Yin C.C., Xie X.D., Jin Y.J., Zhang S.J., Yang B., Ye G.Y., Chen S.Y., Luan W.J. (2019). Overexpression of *OsARD1* Improves Submergence, Drought, and Salt Tolerances of Seedling Through the Enhancement of Ethylene Synthesis in Rice. Front. Plant Sci..

[87] Jin J., Duan J.L., Shan C., Mei Z.L., Chen H.Y., Feng H.F., Zhu J., Cai W.M. (2020). Ethylene insensitive3-like2 (OsEIL2) confers stress sensitivity by regulating *OsBURP16*, the β subunit of polygalacturonase (PG1β-like) subfamily gene in rice. Plant Sci..

[88] Dou L.R., He K.K., Higaki T., Wang X.F., Mao T.L. (2018). Ethylene Signaling Modulates Cortical Microtubule Reassembly in Response to Salt Stress. Plant Physiol..

[89] Wu D., Ji J., Wang G., Guan C., Jin C. (2014). *LchERF*, a novel ethylene-responsive transcription factor from *Lycium chinense*, confers salt tolerance in transgenic tobacco. Plant Cell Rep..

[90] Qin H., Pandey B.K., Li Y.X., Huang G.Q., Wang J., Quan R.D., Zhou J.H., Zhou Y., Miao Y.C., Zhang D.B. (2022). Orchestration of ethylene and gibberellin signals determines primary root elongation in rice. Plant Cell.

[91] Kang J.M., Xie W.W., Sun Y., Yang Q.C., Wu M.S. (2010). Identification of genes induced by salt stress from *Medicago truncatula* L. seedlings. Afr. J. Biotechnol..

[92] Sun X.M., Zhang L.L., Wong D.C.J., Wang Y., Zhu Z.F., Xu G.Z., Wang Q.F., Li S.H., Liang Z.C., Xin H.P. (2019). The ethylene response factor VaERF092 from Amur grape regulates the transcription factor VaWRKY33, improving cold tolerance. Plant J..

[93] Hu Z.R., Huang X.B., Amombo E., Liu A., Fan J.B., Bi A.Y., Ji K., Xin H.P., Chen L., Fu J.M. (2020). The ethylene responsive factor CdERF1 from bermudagrass (*Cynodon dactylon*) positively regulates cold tolerance. Plant Sci..

[94] Ding F., Wang C., Xu N., Wang M.L. (2022). The ethylene response factor SlERF.B8 triggers jasmonate biosynthesis to promote cold tolerance in tomato. Environ. Exp. Bot..

[95] Wang Y.C., Jiang H.Y., Mao Z.L., Liu W.J., Jiang S.H., Xu H.F., Su M.Y., Zhang J., Wang N., Zhang Z.Y. (2021). Ethylene increases the cold tolerance of apple via the MdERF1B-MdCIbHLH1 regulatory module. Plant J..

[96] García M.J., Lucena C., Romera F.J., Alcántara E., Pérez-Vicente R. (2010). Ethylene and nitric oxide involvement in the up-regulation of key genes related to iron acquisition and homeostasis in Arabidopsis. J. Exp. Bot..

[97] Binder B.M., Walker J.M., Gagne J.M., Emborg T.J., Hemmann G., Bleecker A.B., Vierstra R.D. (2007). The Arabidopsis EIN3 binding F-Box proteins EBF1 and EBF2 have distinct but overlapping roles in ethylene signaling. Plant Cell.

[98] Tao J.J., Chen H.W., Ma B., Zhang W.K., Chen S.Y., Zhang J.S. (2015). The Role of Ethylene in Plants Under Salinity Stress. Front. Plant Sci..

[99] Zhang M., Liu S.L., Wang Z., Yuan Y.Q., Zhang Z.F., Liang Q.J., Yang X., Duan Z.B., Liu Y.C., Kong F.J. (2022). Progress in soybean functional genomics over the past decade. Plant Biotechnol. J..

[100] Zheng D.C., Han X., An Y., Guo H.W., Xia X.L., Yin W.L. (2013). The nitrate transporter NRT2.1 functions in the ethylene response to nitrate deficiency in Arabidopsis. Plant Cell Environ..

[101] Catinot J., Huang J.B., Huang P.Y., Tseng M.Y., Chen Y.L., Gu S.Y., Lo W.S., Wang L.C., Chen Y.R., Zimmerli L. (2015). ETHYLENE RESPONSE FACTOR 96 positively regulates *Arabidopsis* resistance to necrotrophic pathogens by direct binding to GCC elements of jasmonate-and ethylene-responsive defence genes. Plant Cell Environ..

[102] Liang Y.S., Ermawati N., Cha J.Y., Jung M.H., Su’udi M., Kim M.G., Ha S.H., Park C.G., Son D. (2010). Overexpression of an AP2/ERF-type Transcription Factor CRF5 Confers Pathogen Resistance to *Arabidopsis* Plants. J. Korean Soc. Appl. Biol. Chem..

[103] Huang Y., Zhang B.L., Sun S., Xing G.M., Wang F., Li M.Y., Tian Y.S., Xiong A.S. (2016). AP2/ERF Transcription Factors Involved in Response to Tomato Yellow Leaf Curly Virus in Tomato. Plant Genome.

[104] Hou X., Wang J.-J., Zhang W.-W., Zhao J.-L., Mao Z.-C. (2024). Cloning of pepper ethylene-responsive proteinase inhibitor Cacl-6468 and its effect on resistance to *Meloidogyne incognita*. Acta Hortic. Sin..

[105] Stotz H.U., Pittendrigh B.R., Kroymann J., Weniger K., Fritsche J., Bauke A., Mitchell-Olds T. (2000). Induced plant defense responses against chewing insects. Ethylene signaling reduces resistance of Arabidopsis against Egyptian cotton worm but not diamondback moth. Plant Physiol..

[106] Ma F.L., Li Z.X., Wang S.Y., Li K.J., Tang F., Jia J.X., Zhao Q.J., Jing P.H., Yang W.Q., Hua C.M. (2023). The F-box protein OsEBF2 confers the resistance to the brown planthopper (*Nilparvata lugens* Stal). Plant Sci..

[107] Yu Z., Duan X., Luo L., Dai S., Ding Z., Xia G. (2020). How Plant Hormones Mediate Salt Stress Responses. Trends Plant Sci..

[108] Steffens B. (2014). The role of ethylene and ROS in salinity, heavy metal, and flooding responses in rice. Front. Plant Sci..

[109] Yang Q.S., Gao J., He W.D., Dou T.X., Ding L.J., Wu J.H., Li C.Y., Peng X.X., Zhang S., Yi G.J. (2015). Comparative transcriptomics analysis reveals difference of key gene expression between banana and plantain in response to cold stress. BMC Genom..

[110] Hu C., Wang M., Zhu C., Wu S., Li J., Yu J., Hu Z. (2024). A transcriptional regulation of ERF15 contributes to ABA-mediated cold tolerance in tomato. Plant Cell Environ..

[111] Zhao R., Xie H., Lv S., Zheng Y., Yu M., Shen L., Sheng J. (2013). LeMAPK4 participated in cold-induced ethylene production in tomato fruit. J. Sci. Food Agric..

[112] Li X., Wilkinson S., Shen J., Forde B.G., Davies W.J. (2017). Stomatal and growth responses to hydraulic and chemical changes induced by progressive soil drying. J. Exp. Bot..

[113] Luo Z. (2006). Hot Water Treatment of Postharvest Mei Fruit to Delay Ripening. HortScience.

[114] Du Z., Gong H., Wang R., Li Y., Wang L., Li B., Li B. (2009). Effect of Hot Water Treatment on Strawberry Fruits Preservation and Its Relationship with Ethylene Gene Expression. Acta Hortic. Sin..

[115] Loayza F.E., Brecht J.K., Simonne A.H., Plotto A., Baldwin E.A., Bai J., Lon-Kan E. (2020). Synergy between hot water treatment and high temperature ethylene treatment in promoting antioxidants in mature-green tomatoes. Postharvest Biol. Technol..

[116] Lynch J., Brown K.M. (1997). Ethylene and plant responses to nutritional stress. Physiol. Plant..

[117] Kou X., Watkins C.B., Gan S.S. (2012). Arabidopsis AtNAP regulates fruit senescence. J. Exp. Bot..

[118] Ma B., Ma T., Xian W., Hu B., Chu C. (2023). Interplay between ethylene and nitrogen nutrition: How ethylene orchestrates nitrogen responses in plants. J. Integr. Plant Biol..

[119] Li S., Wu P., Yu X.F., Cao J.P., Chen X., Gao L., Chen K.S., Grierson D. (2022). Contrasting Roles of Ethylene Response Factors in Pathogen Response and Ripening in Fleshy Fruit. Cells.

[120] Lephatsi M.M., Meyer V., Piater L.A., Dubery I.A., Tugizimana F. (2021). Plant Responses to Abiotic Stresses and Rhizobacterial Biostimulants: Metabolomics and Epigenetics Perspectives. Metabolites.

[121] Wang D., Xie D., Zhang J., Cai B., Yang B., Zhou L., Huang X. (2023). Comprehensive analysis of the coding and non-coding RNA transcriptome expression profiles of hippocampus tissue in tx-J animal model of Wilson’s disease. Sci. Rep..

[122] Esteve-Puig R., Bueno-Costa A., Esteller M. (2020). Writers, readers and erasers of RNA modifications in cancer. Cancer Lett..

[123] Wolny E., Braszewska-Zalewska A., Hasterok R. (2014). Spatial distribution of epigenetic modifications in Brachypodium distachyon embryos during seed maturation and germination. PLoS ONE.

[124] Abdulraheem M.I., Xiong Y., Moshood A.Y., Cadenas-Pliego G., Zhang H., Hu J. (2024). Mechanisms of Plant Epigenetic Regulation in Response to Plant Stress: Recent Discoveries and Implications. Plants.

[125] Ding X., Liu X., Jiang G., Li Z., Song Y., Zhang D., Jiang Y., Duan X. (2022). SlJMJ7 orchestrates tomato fruit ripening via crosstalk between H3K4me3 and DML2-mediated DNA demethylation. New Phytol..

[126] Nottke A., Colaiácovo M.P., Shi Y. (2009). Developmental roles of the histone lysine demethylases. Development.

[127] Francelle L., Lotz C., Outeiro T., Brouillet E., Merienne K. (2017). Contribution of Neuroepigenetics to Huntington′s Disease. Front. Hum. Neurosci..

[128] Zhang F., Wang L., Qi B., Zhao B., Ko E.E., Riggan N.D., Chin K., Qiao H. (2017). EIN2 mediates direct regulation of histone acetylation in the ethylene response. Proc. Natl. Acad. Sci. USA.

[129] Wang L., Zhang F., Qiao H. (2020). Chromatin Regulation in the Response of Ethylene: Nuclear Events in Ethylene Signaling. Small Methods.

[130] Wang L., Qiao H. (2019). New Insights in Transcriptional Regulation of the Ethylene Response in Arabidopsis. Front. Plant Sci..

[131] Xiao Y.Y., Chen J.Y., Kuang J.F., Shan W., Xie H., Jiang Y.M., Lu W.J. (2013). Banana ethylene response factors are involved in fruit ripening through their interactions with ethylene biosynthesis genes. J. Exp. Bot..

[132] Jiang S., Guo C., Zhang W., Che W., Zhang J., Zhuang S., Wang Y., Zhang Y., Liu B. (2019). The Integrative Regulatory Network of circRNA, microRNA, and mRNA in Atrial Fibrillation. Front. Genet..

[133] Wang Y., Wang Q., Gao L., Zhu B., Ju Z., Luo Y., Zuo J. (2017). Parsing the Regulatory Network between Small RNAs and Target Genes in Ethylene Pathway in Tomato. Front. Plant Sci..

[134] Liu Y., Li D., Yan J., Wang K., Luo H., Zhang W. (2019). MiR319 mediated salt tolerance by ethylene. Plant Biotechnol. J..

[135] Yu J., Qiu K., Sun W., Yang T., Wu T., Song T., Zhang J., Yao Y., Tian J. (2022). A long noncoding RNA functions in high-light-induced anthocyanin accumulation in apple by activating ethylene synthesis. Plant Physiol..

[136] Wu S., Wu D., Song J., Zhang Y., Tan Q., Yang T., Yang J., Wang S., Xu J., Xu W. (2022). Metabolomic and transcriptomic analyses reveal new insights into the role of abscisic acid in modulating mango fruit ripening. Hortic. Res..

[137] Shen H., Luo B., Ding Y., Xiao H., Chen G., Yang Z., Hu Z., Wu T. (2024). The YABBY Transcription Factor, SlYABBY2a, Positively Regulates Fruit Septum Development and Ripening in Tomatoes. Int. J. Mol. Sci..

[138] Hartman S., Liu Z.G., van Veen H., Vicente J., Reinen E., Martopawiro S., Zhang H.T., van Dongen N., Bosman F., Bassel G.W. (2019). Ethylene-mediated nitric oxide depletion pre-adapts plants to hypoxia stress. Nat. Commun..

[139] Geigenberger P. (2003). Response of plant metabolism to too little oxygen. Curr. Opin. Plant Biol..

[140] Hattori Y., Nagai K., Furukawa S., Song X.-J., Kawano R., Sakakibara H., Wu J., Matsumoto T., Yoshimura A., Kitano H. (2009). The ethylene response factors *SNORKEL1* and *SNORKEL2* allow rice to adapt to deep water. Nature.

[141] Liu Z., Hartman S., van Veen H., Zhang H., Leeggangers H., Martopawiro S., Bosman F., de Deugd F., Su P., Hummel M. (2022). Ethylene augments root hypoxia tolerance via growth cessation and reactive oxygen species amelioration. Plant Physiol..

[142] Lin C.-C., Chao Y.-T., Chen W.-C., Ho H.-Y., Chou M.-Y., Li Y.-R., Wu Y.-L., Yang H.-A., Hsieh H., Lin C.-S. (2019). Regulatory cascade involving transcriptional and N-end rule pathways in rice under submergence. Proc. Natl. Acad. Sci. USA.

[143] Zhang H., Li A., Zhang Z., Huang Z., Lu P., Zhang D., Liu X., Zhang Z.-F., Huang R. (2016). Ethylene Response Factor TERF1, Regulated by ETHYLENE-INSENSITIVE3-like Factors, Functions in Reactive Oxygen Species (ROS) Scavenging in Tobacco (*Nicotiana tabacum* L.). Sci. Rep..

[144] Xu K., Xu X., Fukao T., Canlas P., Maghirang-Rodriguez R., Heuer S., Ismail A.M., Bailey-Serres J., Ronald P.C., Mackill D.J. (2006). *Sub1A* is an ethylene-response-factor-like gene that confers submergence tolerance to rice. Nature.

[145] Wu L., Zhang Z., Zhang H., Wang X.-C., Huang R. (2008). Transcriptional Modulation of Ethylene Response Factor Protein JERF3 in the Oxidative Stress Response Enhances Tolerance of Tobacco Seedlings to Salt, Drought, and Freezing. Plant Physiol..

[146] Peng J., Li Z., Wen X., Li W., Shi H., Yang L., Zhu H., Guo H. (2014). Salt-Induced Stabilization of EIN3/EIL1 Confers Salinity Tolerance by Deterring ROS Accumulation in *Arabidopsis*. PLoS Genet..

